# Combination Drug Therapy for Pain following Chronic Spinal Cord Injury

**DOI:** 10.1155/2012/840486

**Published:** 2012-03-18

**Authors:** Aldric Hama, Jacqueline Sagen

**Affiliations:** The Miami Project to Cure Paralysis, Miller School of Medicine, University of Miami, 1095 SW 14th Terrace, Miami, FL 33136, USA

## Abstract

A number of mechanisms have been elucidated that maintain neuropathic pain due to spinal cord injury (SCI). While target-based therapeutics are being developed based on elucidation of these mechanisms, treatment for neuropathic SCI pain has not been entirely satisfactory due in part to the significant convergence of neurological and inflammatory processes that maintain the neuropathic pain state. Thus, a combination drug treatment strategy, wherein several pain-related mechanism are simultaneously engaged, could be more efficacious than treatment against individual mechanisms alone. Also, by engaging several targets at once, it may be possible to reduce the doses of the individual drugs, thereby minimizing the potential for adverse side effects. Positive preclinical and clinical studies have demonstrated improved efficacy of combination drug treatment over single drug treatment in neuropathic pain of peripheral origin, and perhaps such combinations could be utilized for neuropathic SCI pain. At the same time, there are mechanisms that distinguish SCI from peripheral neuropathic pain, so novel combination therapies will be needed.

## 1. Introduction

Tissue injury or disease may lead to a persistent pain state. Chronic pain is maintained through a combination of neural and nonneural mechanisms operating simultaneously at peripheral and central nervous systems [1, 2]. At the site of peripheral tissue damage, a number of inflammatory mediators are released that activate and recruit immune cells, initiating the process of tissue repair. Also, many of these mediators released from recruited immune cells, including excitatory amino acids, neuropeptides, and cytokines, sensitize primary afferent nociceptors [3–5]. The persistent pain state could be maintained, in part, by the overproduction of these mediators and by the overexpression by genes of cell membrane-bound proteins, such as receptors and ion channels, and intracellular signaling complexes in peripheral nerves [6–8]. Furthermore, the physiological responses of spinal dorsal horn neurons and primary afferent neurons are permanently altered, such that their responses to peripheral, cutaneous stimulation are exaggerated and persist long after the application of stimulation. These physiological changes are believed to be maintained by long-lasting changes in genes and, akin to the process observed in peripheral nociceptors, overexpression of membrane-bound proteins and overactivation of intracellular signaling [9, 10].

Spontaneous activity has been demonstrated from injured peripheral sensory neurons and from CNS neurons, proximally and distally to the site of injury [11–13]. The abnormal neurophysiological responses to peripheral injury, sensitization, and spontaneous activity are believed to be the neural basis of tissue injury-induced chronic pain, which is characterized by cutaneous hypersensitivity and spontaneous pain.

In spinal cord injury (SCI), spontaneously active CNS neurons, found spinally and supraspinally, have been documented in both clinical and experimental settings [14–20]. Experimental evidence suggests that reducing the activity of these neurons leads to a decrease in chronic pain symptoms. Drugs that have demonstrated to decrease CNS neural activity, including opioids, *γ*-aminobutyric acid (GABA) receptor agonists, and sodium channel blocking drugs, are antinociceptive in animal pain models and these drugs are also analgesic in clinical pain states [21]. The data suggest that robust pain relief can be obtained though suppressing abnormal neural activity. Obtaining direct evidence, such as electrophysiological and neurochemical, of drug effects from patients as performed in animals, is a tremendous technical hurdle. However, noninvasive imaging may be an alternate method to quantify drug effects on brain neurochemistry and activity and these data could correlate with pain relief [22, 23]. Furthermore, such data could be used to guide drug discovery.

The existence of multiple, often overlapping mechanisms that have been identified so far not only underscores the biological complexity of chronic pain, but the difficultly in providing significant pain relief with currently available analgesic pharmacotherapies. The vast array of pain-related mechanisms, however, invites development of a nearly endless list of potential treatments, especially treatments that target more than one mechanism.

## 2. Combination Therapy: Synergism

One could take advantage of the parallel activities of molecular targets by engaging several of these targets at once, wherein the goal is a combination treatment that is superior to that of individual target-specific treatments [24, 25]. The concept of synergism has been demonstrated in the clinical setting for a variety of indications such as the treatment of cancer and infections [26]. Combining drugs may lead to either additive or non-additive effects. If the effect of a combination of two or more drugs does not significantly deviate from the theoretical or expected effect based on their individual dose-response curves, then the effect of the combination is said to be additive. There are two types of nonadditive effects that may result with combination treatment. First, if the effect of the combination is greater than expected, then the combination is said to be superadditive, or synergistic. The total dose of the synergistic combination can be lower than what would be expected from the individual dose-response curves, which may also diminish the risk of adverse side effects associated with either drug. Secondly, the total dose needed to induce a certain effect may be higher than what is expected. In this case, the combination is said to be subadditive or antagonistic.

The key is that the experimentally derived result be statistically significant from the expected result. One method to determine this is by isobolographic analysis, wherein the doses of the constituents that give, for example, a 50% antinociceptive effect, are plotted on the *x*-axis and *y*-axis [26, 27]. The line connecting these points is said to be the “line of additivity,” a locus of dose pairs of the constituents expected to demonstrate equal antinociception (“zero interaction”). The amount of each constituent in the combination can be based on the relative potency of the constituents. Following construction of a dose-response curve of the combination, the 50% antinociceptive dose is determined and statistical analysis is used to determine whether or not the effect of the combination significantly deviates from zero interaction, whether the effect of the combination is either synergistic, antagonistic, or merely additive. While one ratio of the constituents may be merely additive, other ratios may lead to either synergistic or antagonistic effects [28]. Thus, the lack of synergy for a given combination should not automatically exclude that combination from further consideration—perhaps other combination ratios could lead to synergy.

There are cases of synergism in which one of the drugs in a two-drug combination demonstrates no efficacy [29]. To demonstrate synergism, a statistically significant increase in the potency of the active drug of the combination compared to the active drug alone is required. Thus, drugs that do not demonstrate efficacy on their own may still be useful when given with drugs that do demonstrate efficacy [30–32]. The advantage of this type of combination is similar to that of a combination in which both drugs demonstrate efficacy—the dose of the efficacious drug in the combination is decreased thereby reducing the potential for adverse side effects.

## 3. Neuropathic Spinal Cord Injury Pain

In the U.S., there are an estimated 256,000 SCI patients and there are approximately 12,000 new cases of SCI each year, most commonly due to motor vehicle accidents and falls [33]. As medical breakthroughs increase life expectancy in general, there will be a growing population of elderly, and life expectance of current SCI patients could increase as well. In addition, with increasing numbers of older persons, it is anticipated that they may develop SCI, due to, for example, accidents [34, 35]. Older SCI, as well as non-SCI, patients are more likely than younger people to report chronic pain [36]. Thus, with the significant expansion of the elderly population in the U.S. and other industrialized countries projected by midcentury and the potential for an increased number of elderly SCI patients, there is an urgent need to develop effective pain therapeutics [37, 38].

In addition to significant losses in motor and visceral functionalities, intractable pain may also result following SCI, a majority of SCI patients reporting the severity of pain as either moderate or severe, such that there is greatly diminished mood and motivation to participate in rehabilitation programs and social interaction [39–41]. Pain may be localized in dermatomes either above, at, or below the level of injury [42–44]. Interestingly, below-level pain has been described as “burning” or “shooting” and accompanied by cutaneous hypersensitivity, symptoms that are characteristic of peripheral neuropathic pain [44, 45]. There is also the possibility of “autonomic dysreflexia,” a condition in which noxious somatic or visceral stimulation below the level of SCI could lead to an acute, uncontrolled sympathetic nervous system response, including a life-threatening increase in blood pressure and tachycardia [46]. Thus, treatments that are tolerated in other pain populations may not be suitable for SCI patients. For example, visceral distention by opioids and antidepressants such as amitriptyline could lead to autonomic dysreflexia.

Furthermore, treatments that are efficacious in peripheral neuropathic pain do not appear to demonstrate the same level of efficacy in neuropathic SCI pain. Amitriptyline, for example, in addition to adverse side effects in SCI patients such as urinary retention, does not appear to be as effective in neuropathic SCI pain as it is in peripheral neuropathic pain [47]. Mexiletine, an orally active analogue of the sodium channel blocking drug lidocaine, also does not show the level of efficacy in SCI patients that has been demonstrated in peripheral neuropathic pain patients [48, 49]. Perhaps the lack of efficacy across patient populations could be due in part to varied testing protocols and outcome measurements in the clinical trials, but some of the differential efficacy could also be due to underlying differences in pain mechanism. If standard pharmacotherapies, when given alone, do not demonstrate efficacy, then combination drug therapy could be a viable option for SCI pain patients.

## 4. Preclinical Combination Drug Therapy in the SCI Rat

To facilitate the evaluation of novel pharmacotherapies for potential clinical use, a rat model of chronic neuropathic SCI pain was recently developed [50, 51]. Four weeks following a brief midthoracic spinal compression, a massive infiltration of monocytes and a robust gliotic response were observed at the injury site. In addition, syrinxes were observed extending for several segments from the injury site. The histopathological findings are reminiscent of that reported following an acute spinal contusion, a widely used method of inducing SCI in rats, and in clinical SCI [52, 53]. Despite significant bilateral motor dysfunction below the level of the injury, similar in degree and extent to that following a contusion injury, rats were responsive to cutaneous stimulation. The methods of quantifying below-level cutaneous hypersensitivity used were the same as those commonly used in rat models of peripheral nerve injury [54, 55]. A long-lasting below-level hypersensitivity to cutaneous stimuli, as observed in clinical SCI pain, was obtained beginning one week following spinal compression, which lasted for at least 12 weeks after-injury [56].

A variety of clinically used analgesic drug were assessed in these rats. The anxiolytic drug diazepam did not demonstrate antinociceptive efficacy, indicating that sedative or muscle relaxant property is not sufficient to ameliorate pain-related behaviors [57, 58]. While mexiletine and the anticonvulsant drug carbamazepine were found to be efficacious in other chronic pain models, these did not demonstrate significant effects in SCI rats [59, 60]. The anti-inflammatory cyclooxygenase-2 selective inhibitor rofecoxib and the nonselective cyclooxygenase inhibitor naproxen also did not affect below-level cutaneous hypersensitivity, suggesting that prostaglandins are not a critical factor in maintaining the neuropathic state in these rats [61]. The doses of rofecoxib and naproxen tested in SCI rats were efficacious, however, in rat models of inflammation-induced pain. The lack of efficacy of several analgesics in SCI rats compared to other rat pain models suggests fundamental differences in mechanisms among the chronic pain states.

At the same time, several drugs that demonstrated antinociception in peripheral neuropathic pain models also demonstrated efficacy in the current SCI model, suggesting that both peripheral and SCI pain share a few common mechanisms. Clinical drugs that are generally characterized as selective for a particular target, including opiates, the GABA_B_ receptor agonist baclofen, the voltage-gated calcium channel (VGCC) blocker gabapentin, and noncompetitive *N*-methyl-D-aspartate (NMDA) receptor antagonists such as ketamine, showed efficacy in both models of SCI and peripheral nerve injury pain [60, 62–66]. The analgesic tramadol also demonstrated efficacy in both SCI and peripheral neuropathic pain models [66–69]. Interestingly, the efficacy of tramadol is likely to be mediated by a combination of several mechanisms: its metabolite is a *μ*-opioid receptor agonist and the drug itself increases synaptic levels of analgesic neurotransmitters serotonin and norepinephrine by blocking the reuptake of these neurotransmitters [69]. Both separate and simultaneous activation of rat spinal cord dorsal horn *μ*-opioid and serotonergic receptors and *α*-adrenoceptors leads to marked antinociception in acute pain tests [70, 71]. Since drugs are usually dosed systemically, antinociceptive effects could be the result of engaging pain-related targets in several sites within the CNS and those targets may also be found in peripheral nerves [72–74]. Given the presence of a number of pain-related mechanisms involved in modulating pain perception, there are potentially numerous combinations that may lead to synergistic analgesia [75].

Some of the common side effects observed with analgesic drugs with systemic bioavailability include somnolence, sedation, dizziness, and nausea [41]. Adverse side effects are inevitable since most currently available analgesics freely distribute throughout the CNS. With combination drug therapy, it may be possible to significantly reduce the incidence of side effects by reducing the doses of the constituents. Alternatively, there are drug delivery methods which may further minimize the incidence of side effects. One method is to deliver drugs into the intrathecal (i.t.) space of the spinal cord [76]. Some analgesics that demonstrated efficacy when given systemically also demonstrated robust efficacy following i.t. injection in SCI rats, indicating that the spinal dorsal horn is a key site of action of these drugs [77–79]. In rats with a spinal hemisection, blockade of spinal NMDA receptors at the site of spinal injury with i.t. injection of the competitive NMDA receptor antagonist AP-5 ameliorated below-level hypersensitivity to innocuous mechanical stimulation (but not injury-induced heat hypersensitivity) [80]. The effects of clinically used NMDA receptor antagonists, such as ketamine or memantine, were not evaluated in these rats. While block of dorsal horn NMDA receptors in general leads to an antinociceptive effect in chronic pain models, it appears that the degree of efficacy depends on whether the antagonist used is a competitive or noncompetitive antagonist [81, 82]. In-house data indicates that i.t. ketamine, at doses that do not induce hind limb dysfunction, does not ameliorate below-level cutaneous hypersensitivity in rats with a spinal compression injury. In contrast, systemic NMDA receptor antagonist treatment is effective, suggesting that supraspinal NMDA receptors have a prominent role in maintaining below-level cutaneous hypersensitivity in spinal compression-injured rats [50]. The main disadvantage of systemic NMDA receptor antagonists, however, is the overlap in doses that lead to significant supraspinally mediated psychomimetic effects and those that lead to antinociception [83]. Even though i.t. ketamine alone was not efficacious, as noted earlier, it could be effective if combined with other i.t. delivered analgesic drugs.

A number of combination therapies have been evaluated for efficacy in preclinical models of neuropathic pain but few have been tested specifically in a preclinical model of neuropathic SCI pain and so their potential clinical usefulness is unknown [24]. Because of possible differences in mechanism between peripheral and SCI pain states, combinations that maybe useful in one state might not show efficacy in the other state. Therefore, it will be crucial to test potential combination drug therapies in models of SCI pain.

Recently, a number of drug combination therapies have been evaluated for antinociceptive efficacy in rats with spinal compression injury. While baclofen is approved for use in spasticity, it has been used off label for the treatment of neuropathic pain, including neuropathic SCI pain [84, 85]. Because systemic dosing can lead to side effects such as sedation and muscle weakness, i.t. baclofen has been utilized as a means of long-term pain treatment. However, pharmacological tolerance to the beneficial effect of i.t. baclofen has been reported, and potentially dangerous withdrawal symptoms may occur if i.t. infusion is suddenly interrupted [86–88]. One method of reducing the dose of baclofen and reducing the possibility of tolerance and the severity of withdrawal is to combine it with other drugs. Preliminary data from SCI rats indicates that i.t. injection of a combination of ketamine and a 50% efficacious dose of baclofen leads to a significant enhancement of baclofen antinociception (unpublished data). The combination also underscores a mechanistic hypothesis, that chronic SCI pain is maintained by a simultaneous decrease in GABAergic inhibition and increase in NMDA receptor-mediated excitation [80, 89, 90]. Therefore, considerable pain relief could be obtained by blocking the NMDA receptor and activating GABA receptors. While ketamine was synergistic with baclofen, combination i.t. treatment of GABA_A_ receptor agonist muscimol and ketamine did not lead to synergism. That there was synergism with GABA_B_, but not with GABA_A_, receptors is puzzling. The lack of synergism could be explained via a paradoxical in vitro finding that activation of the GABA_A_ receptor leads to the activation of NMDA receptors [91]. Thus, there is the need for further elaboration of possible interactions between pain-related targets in order to find useful combinations for clinical efficacy. Given all of the potential interactions within the pain transmission system, it appears that synergism is a novel occurrence at best [29].

Ziconotide, a synthetic analogue of a peptide derived from the marine snail *Conus magnus*, is prescribed for use as an i.t. monotherapeutic for severe chronic pain [92]. Preclinical and limited clinical studies indicate that ziconotide may be useful in below-level SCI pain [93, 94]. Ziconotide acts via blockade of the N-type VGCC, which are expressed on central terminals of primary afferent nociceptors, which synapse with dorsal horn spinal neurons [95]. Blocking spinal N-type VGCCs prevents an influx of calcium ions and the subsequent increase in intracellular calcium concentration, thereby preventing the calcium-mediated release of neurotransmitter from central terminals and transmission of nociceptive signaling across the synapse. N-type VGCC found on spinal neurons are also blocked by ziconotide, thereby reducing nociceptive signaling within the CNS. Furthermore, N-type VGCCs in the “neuropathic state” appears to be more sensitive to ziconotide block compared to N-type channels from uninjured animals, since treatment with ziconotide does not affect nociception in uninjured animals [96, 97]. There are reports of psychiatric effects with i.t. ziconotide treatment, which are ameliorated when the dose is lowered, indicating that the side effects are target mediated [98]. Another naturally derived peptide, conantokin-G, blocks NMDA receptors, with an antinociceptive effect similar to that of small molecule NMDA receptor antagonists [99]. Interestingly, i.t. treatment with conantokin-G in rats does not lead to the characteristic side effects typically observed with small molecule NMDA receptor antagonists, so this peptide could find potential use as a monotherapeutic. Nonetheless, the i.t. combination of ziconotide and conantokin-G leads to a synergistic antinociception in SCI rat, whereas the combination of the two leads to additive antinociception in other rat pain models [93]. Why synergism of this combination is observed in SCI compared to other injury states is not entirely clear at this point. There are a number of naturally derived substances, other than peptides, that block ion channels and receptors which may confer significant clinical analgesia alone and which may also significantly enhance the analgesic efficacy of currently available drugs [100, 101].

Cannabinoids have been used for hundreds, if not thousands, of years as a therapeutic for a variety of conditions, including pain [102]. Cannabinoid (CB) receptor agonists have demonstrated robust antinociceptive effects in a variety of preclinical models of chronic pain [103, 104]. Activation of CB receptors alone and in combination with other receptors involved in nociceptive processing leads to synergistic antinociception in rodent pain models [105–107]. There are varying degrees of efficacy of CB receptor ligands in a number of clinical pain states, although they are not as robust as reported in preclinical studies [108]. The mixed levels of clinical efficacy could be due in part to pharmacokinetics. Efficacy has been reported with inhaled CB receptor ligands but not for orally ingested CB receptor ligands in central pain states [49, 109, 110]. A problem that arises from systemic delivery of CB receptor agonists is activation of both spinal and supraspinal receptors, which not only leads to antinociception but also significant psychomotor effects [111]. Given the strong psychomotor side effects observed with therapeutic doses of CB1 receptor agonist, and the sociopolitical controversy surrounding the use of this class of drug for medical use in general, alternative means by which to engage CB receptors for pain relief are needed.

One method to indirectly engage CB receptors would be to increase synaptic concentrations of endogenous cannabinoid receptor ligands (or “endocannabinoids”), such as anandamide (*N*-arachidonoylethanolamide), by inhibiting the enzyme which degrades it, fatty acid amide hydrolase (FAAH). Other enzymes involved in metabolizing other endocannabionods could also be utilized as pain targets [112]. Antinociceptive effects have been demonstrated by increasing CNS anandamide with FAAH inhibitors in preclinical models of neuropathic and inflammatory pain, and the effects were CB receptor dependent [113–115]. Furthermore, the antinociceptive effects were not accompanied by the adverse side effects commonly observed with efficacious doses of small molecule CB receptor agonists. Development of selective and potent FAAH inhibitors for clinical use is currently on-going. However, a metabolite of the over-the-counter drug acetaminophen (acetyl-para-aminophenol), AM404, has been shown to increase synaptic levels of anandamide by blocking the neuronal reuptake of anandamide [116]. Acetaminophen itself acts through various mechanisms and these mechanisms in total, including its effect on synaptic endocannabinoid levels, could explain its analgesic effects [117]. Acetaminophen is safe when taken as directed, and has a long clinical history, either alone or as a combination therapeutic [118, 119]. Until recently, acetaminophen-based combination drug therapy has not been evaluated in SCI pain models [120]. Acetaminophen alone did not alter below-level cutaneous hypersensitivity, even at doses that demonstrated efficacy in other pain models. However, combining acetaminophen with a 50% efficacious dose of gabapentin significantly increased the efficacy of gabapentin. In addition, the antinociception was attenuated with treatment of the CB1 receptor antagonist rimonabant but not the CB2 receptor antagonist AM630, indicating that the combination antinociceptive effect is mediated through endocannabinoids activating CB1 receptors (the antinociceptive effect of gabapentin alone was not blocked with pretreatment of rimonabant). The combination of acetaminophen with morphine was also synergistic and partially mediated by CB1 receptors. Not all acetaminophen combinations demonstrated synergism, however, as combination with either memantine or tramadol were merely additive. These results suggest that acetaminophen combinations could be useful in clinical SCI pain by combining indirect CB receptor activation with modulation of other pain-related targets. In addition, increasing endocannabinoids could be a method to circumvent the use of potent CB receptor agonists which lead to supraspinally mediated side effects. In practice, there may be a period of trail and error in determining an optimal combination that will lead to synergism in humans.

Potentially useful combinations are not always available in convenient oral formulation and this may hamper patient compliance. The possibilities of adverse drug interactions and, for i.t. administration, tissue toxicity need to be carefully considered. As a potential alternative to pharmacotherapeutics, transplantation of cells that release a mixture of analgesic substances could be a long-term means to reduce neuropathic SCI pain. A number of studies have demonstrated that adrenal medullary chromaffin cells, which release catecholamines, opioid peptides, other neuropeptides including the NMDA receptor antagonist histogranin, and neurotrophic factors, implanted in the spinal subarachnoid lumbar space, lead to significant antinociception in various animal models of pain [121, 122]. Because of the difficulty of obtaining cadaver adrenal medullary tissue for human implantation, cell lines have been engineered to secrete analgesic neurotransmitters, such as GABA and serotonin [123]. The genes for neuroactive peptides, such as ziconotide and histogranin, could be inserted into the genome of these cells [124, 125]. Thus, a mixture of substances would be continuously released into the CSF to ameliorate pain on a long-term basis, without the need for maintenance or refilling the reservoir of an i.t. drug infusion pump. An added advantage is that these mixtures could be designed to reduce the potential for analgesic tolerance. The addition of NMDA receptor antagonists, for example, appears to delay or inhibit the onset of tolerance to the antinociceptive effects of opioids that emerges over time when they are given alone in animals [126].

## 5. Clinical Use of Combination Drug Therapy in SCI Pain

The preclinical data suggests that clinically used drugs in combination could be useful in ameliorating SCI pain. Even drugs that do not demonstrate efficacy alone could still be useful if combined with a drug that is known to offer pain relief. While the preclinical data are indeed promising, few clinical studies have been performed and fewer still have demonstrated analgesic synergism on the order of magnitude observed in preclinical studies. Ideally, the demonstration of synergism should be carried out with methodological rigor similar to that performed in preclinical studies, such as generation of dose-effect curves of the constituents alone and a dose-effect curve of the combination, the proportion of the constituents of the combination determined by the individual curves. Also, each drug alone and in combination would be compared with a placebo treatment group [25, 127]. The presence of genuine synergism is further complicated by the fact that few, if any, studies suspend analgesic usage prior to the commencement of the study, such that the supra-additive effect of the drugs under investigation could be due to nonstudy medications. Given the limited number of suitable clinical subjects and ethical reasons, analgesic synergism studies are few and far between. Nonetheless, a few carefully designed studies have demonstrated synergism in the clinical setting. One study demonstrated a synergistic interaction between i.t. morphine and clonidine, an *α*_2_-adrenoceptor agonist, in SCI patients [128]. Doses of i.t. morphine and clonidine were titrated in each patient until either efficacy or side effects was obtained. A 50% efficacious dose of each drug was calculated, then given as a mixture, which yielded analgesia greater than either drug alone and equally analgesic in SCI patients with either at-level or below-level neuropathic pain.

Other studies evaluated the effect of a second therapeutic as an “add-on,” wherein the second drug is added to an already existing drug treatment. For example, i.t. morphine was evaluated as an add-on in SCI patients who were undergoing treatment with a stabilized dose of i.t. baclofen for pain and spasticity [129]. Although most of the patients who received add-on i.t. morphine tolerated the combination, the average reduction in pain was modest, about 35%. A case report noted improved pain and spasm relief in a SCI patient with clonidine added to i.t. baclofen [130]. Prior to the addition of clonidine, the patient found it necessary to escalate the dose of baclofen needed for pain relief over time. Two years following the initiation of the combination therapy, no further increase in the dose of baclofen was necessary. Ziconotide has also been used as an add-on to i.t. baclofen (and, alternatively, baclofen as an add-on to i.t. ziconotide) and in combination with i.t. hydromorphone, an opioid [94, 131].

One novel combination evaluated in neuropathic SCI pain patients was i.v. ketamine (given once a day for seven days) as an add-on to oral gabapentin, compared to i.v. saline treatment and oral gabapentin (300 mg TID) [132]. During the week of i.v. treatment, the ketamine add-on group showed markedly lower average pain scores compared to the saline-treated group. Furthermore, the group that received i.v. ketamine continued to show reductions in pain scores for at least two weeks after the last infusion of ketamine. After this period, the pain scores of the ketamine-treated group were similar to that of the saline-treated group. This study also demonstrated two interesting effects of oral gabapentin treatment in SCI patients. First, in both groups, pain scores at the end of the study (five weeks in duration) were reduced to about half that of baseline, pretreatment pain scores. Thus, the study confirmed the persistent analgesia obtained with regular gabapentin treatment in SCI patients [41]. Second, in the i.v. saline-treated group, a significant analgesic effect with oral gabapentin treatment can be discerned on the first day of treatment, and analgesia improved over the course of gabapentin treatment. An acute analgesic effect of oral gabapentin has also been reported in clinical peripheral neuropathic pain, reducing both spontaneous pain and cutaneous hypersensitivity within hours of treatment [133]. The mechanism of the two-week analgesic enhancement following ketamine treatment is unknown. It is possible that other combinations with gabapentin may lead to an enhanced and persistent analgesia.

Dose-dependent effects of the add-on drug or the on-going therapeutic were not established in these studies. Perhaps the effect of the combinations was derived mainly from the add-on drug rather than the combination per se. Clearly, further studies are needed to determine if these combinations fulfill the definition of synergism and what the optimal drug ratio would be, but concurrent activation of spinal GABA_B_ receptors with either N-type VGCC block, *μ*-opioid receptor, or *α*_2_-adrenoceptor activation could be a potentially therapeutic combination. Currently, the only drugs that are approved by the U.S. Food and Drug Administration for i.t. in humans are ziconotide, morphine, and baclofen, and no recommendation has been issued regarding the mixture of these drugs for intrathecal use [134]. Furthermore, the safety of other unapproved drugs for i.t. use has not been extensively determined.

While it may be relatively straightforward for some SCI patients to take medications orally, other patients may have difficulty swallowing. In the case where i.t. drug delivery may not also be an option, topical drug application may be warranted. At-level neuropathic SCI pain is hypothesized to result from the sensitization of primary afferent nociceptors due to the trauma that led to SCI [42]. Alternatively, centrally mediated processes resulting from SCI feedback onto central terminals of nociceptors, which in turn leads to nociceptor sensitization [135]. Activation of nociceptors in some SCI patients with topical capsaicin leads to increased hypersensitivity to cutaneous stimulation and a burning painful sensation [136]. This indicates that not only are at-level nociceptors intact, but normal functionality has been significantly altered. Application of lidocaine patches in the painful dermatome reduces spontaneous and evoked pain. Other topical treatments have been tested in peripheral neuropathic pain and perhaps these could be used, either alone or in combination, for at-level neuropathic SCI pain [137–139]. It is not known if topical treatment would be effective on below-level SCI pain. A significant peripheral contribution to SCI pain suggests that targeting nociceptors could be an effective treatment and that the drug (or combination of drugs) does not have to enter the CNS, thereby circumventing the problem of CNS-mediated adverse side effects [74, 140, 141].

The contribution of peripheral nociceptors in neuropathic SCI pain, however, could vary between patients. A clinical report was unable to demonstrate a change of peripheral nociceptor responsiveness to capsaicin treatment, either at, below, or above the lesion, in SCI patients [142]. This result suggests that in some patients, central processes, rather than functional changes in peripheral nociceptors, maintain neuropathic SCI pain. Thus, treatments that focus on attenuating the abnormal neural activity at spinal and supraspinal levels would benefit these patients. Perhaps a topical capsaicin test could be used to assess peripheral nociceptor functionality and based on the result, tailor treatment for that patient. A pressing challenge for health care providers will be to identify the relevant mechanism in each SCI patient such that therapeutic intervention will address those particular mechanism and yield pain relief.

## 6. Other Possible Combination Treatments

One other instance of synergy demonstrated in animals, which may not have immediate clinical applicability, is injection of a drug at different sites of the neuraxis [143, 144]. Such “autosynergy” suggests an interaction of two or more neural sites are required for the analgesic effect of a given drug and that loss or dysfunction of one site will result in decreased efficacy of that drug. This concept could be applied to the use of electrical stimulators implanted in CNS regions involved in nociceptive processing [145]. Neither deep brain stimulation nor spinal cord (dorsal column) stimulation in SCI patients have demonstrated efficacy on pain, but perhaps the combination of the two, spinal and supraspinal or into distinct but complementary brain nuclei, could lead to robust analgesia [146, 147]. Furthermore, drugs, either systemic or i.t., could also be combined with stimulators, to enhance the effect of the stimulator or vice versa [148, 149]. Thus, the application of synergism may not be limited to pharmacotherapeutics.

## 7. Conclusion

Combination drug therapy could fulfill current needs in at least two areas. It is foreseen that noteworthy new treatments will emerge in the near future with the increased elucidation of the mechanism that underlies neuropathic SCI pain. However, the discovery process involving novel therapeutic targets is both expensive and highly time consuming, and that the safety of treatments based on those targets will not be clear until the completion of extensive human trials [150]. Until the day when novel treatments are ready for widespread use, patients could be treated with currently available pharmacotherapies with known biological and safety profiles in novel combinations. Soon-to-be-initiated clinical studies will evaluate the suitability of cellular transplants to repair SCI and to promote functional recovery. However, there is the possibility that transplantation will induce sprouting of afferent central terminals, such that SCI patients who have not experienced pain may begin to experience it or that on-going pain in other patients will worsen [151–154]. Again, combination drug therapy could be used as these patients undergo transplantation treatment.

There are challenges that will need to be addressed with combination drug therapy for SCI pain, similar to the challenges noted for other patient populations, including timing of drug dosing and dose ratio [25]. Currently, the emergence and submergence of particular pain-related mechanisms over time are not well delineated, and it is assumed that many of the processes occur all at once. Aging may alter the temporal aspect of pain mechanisms which could in turn alter responsiveness to therapeutics [155–158]. Perhaps greater efficacy and safety could be obtained if drugs are combined at defined times during the course of treatment. With greater understanding of the temporal aspects of pain mechanisms, irrelevant drugs can be excluded depending on the duration of the pain symptoms. As mentioned earlier, the ratio of constituents in a combination could also change, depending on the prevalence or robustness of a particular mechanism [65, 68]. Thus, greater understanding of post-injury mechanism timing will be needed.

Finally, although animal models have been useful in elaborating the in vivo neurological and biochemical mechanisms of pain, one limitation is the difficulty of obtaining spontaneous or unevoked measures of pain. As pain involves an affective as well as sensory component, the effect of novel analgesics on this component is currently unknown, and may be as therapeutically important as reducing the somatosensory component of pain. It is clear that below-level cutaneous stimulation in rats leads to pain-related behaviors such as vocalization and licking and biting of the stimulated area, indicating a supraspinally mediated component [159, 160]. In fact, such an overlap, between cutaneous hypersensitivity and below-level pain in SCI patients is clinically observed [56]. Given the significant contribution of supraspinal structures, including cortical structures, in pain, models of integrated, “purposeful” behaviors in animal pain models have been proposed [161–163]. There are neuroanatomical and cognitive issues that will need to be addressed in tying complex behavior in nonhuman species to human behavior, which should be made clear when drawing conclusions from such behavioral models [164–166]. As with other preclinical models, the responses to pharmacological manipulation, to both analgesics and nonanalgesics (as “active placebos”), will need to be elaborated. Testing in alternate species and evaluating spontaneous behavior could also prove highly useful in closing the gap between laboratory proof-of-concept and utilizing discoveries in the clinic [167].

## 8. Summary

The benefits of combination drug therapy for the treatment of neuropathic SCI pain include potential analgesic synergism, wherein the efficacy of the combination is significantly greater than that of the constituents alone and deceased potential for adverse side effects. Recent advances in the neurosciences have uncovered numerous pain-related molecular targets. However, neuropathic SCI pain remains difficult to treat with available pharmacotherapeutics since they do not specifically address neuropathic SCI pain. Engaging more than one relevant target via combination drug therapy may significantly improve pain management in SCI patients. Further clinical studies will be needed to address key issues such as identifying which target combinations could yield the most robust efficacy and the optimal dose ratio of a given combination drug therapy.
